# Bovine tuberculosis at a cattle-small ruminant-human interface in Meskan, Gurage region, Central Ethiopia

**DOI:** 10.1186/1471-2334-11-318

**Published:** 2011-11-15

**Authors:** Rea Tschopp, Kidist Bobosha, Abraham Aseffa, Esther Schelling, Meseret Habtamu, Rahel Iwnetu, Elena Hailu, Rebuma Firdessa, Jemal Hussein, Douglas Young, Jakob Zinsstag

**Affiliations:** 1 Swiss Tropical and Public Health Institute, PO Box, CH-4002, Basel, Switzerland; 2 Armauer Hansen Research Institute (AHRI/ALERT), PO Box 1005, Addis Ababa, Ethiopia; 3 Department of Microbiology, Imperial College London, South Kensington Campus London, SW7 2AZ, UK

## Abstract

**Background:**

Bovine tuberculosis (BTB) is endemic in Ethiopian cattle. The aim of this study was to assess BTB prevalence at an intensive contact interface in Meskan Woreda (*district*) in cattle, small ruminants and suspected TB-lymphadenitis (TBLN) human patients.

**Methods:**

The comparative intradermal test (CIDT) was carried out for all animals involved in the cross-sectional study and results interpreted using a > 4 mm and a > 2 mm cut-off. One PPD positive goat was slaughtered and lymph nodes subjected to culture and molecular typing. In the same villages, people with lymphadenitis were subjected to clinical examination. Fine needle aspirates (FNA) were taken from suspected TBLN and analyzed by smear microscopy and molecular typing.

**Results:**

A total of 1214 cattle and 406 small ruminants were tested for BTB. In cattle, overall individual prevalence (> 2 mm cut-off) was 6.8% (CI: 5.4-8.5%) with 100% herd prevalence. Only three small ruminants (2 sheep and 1 goat) were reactors. The overall individual prevalence in small ruminants (> 2 mm cut-off) was 0.4% (CI: 0.03-5.1%) with 25% herd prevalence. Cattle from owners with PPD positive small ruminants were all PPD negative. 83% of the owners kept their sheep and goats inside their house at night and 5% drank regularly goat milk.

FNAs were taken from 33 TBLN suspected cases out of a total of 127 screened individuals with lymph node swellings. Based on cytology results, 12 were confirmed TBLN cases. Nine out of 33 cultures were AFB positive. Culture positive samples were subjected to molecular typing and they all yielded *M. tuberculosis*. *M. tuberculosis *was also isolated from the goat that was slaughtered.

**Conclusions:**

This study highlighted a low BTB prevalence in sheep and goats despite intensive contact with cattle reactors. TBLN in humans was caused entirely by *M. tuberculosis*, the human pathogen. *M. tuberculosis *seems to circulate also in livestock but their role at the interface is unknown.

## Background

Bovine tuberculosis (BTB) caused by *Mycobacterium bovis*, a Gram-positive acid-fast bacterium and close member of the *Mycobacterium Tuberculosis *Complex (MTC) has been described in various domestic and wild mammals as well as in humans around the world [1-4].

People can get infected through consumption of raw animal products and/or close contact with infected animals, particularly livestock [5,6]. More over, BTB is an economical burden through loss in animal productivity, cost of control and eradication programs, and loss in trade markets [7]. Control and eradication programs are targeting cattle population. The World Organization for Animal Health (OIE) published guidelines and standards for BTB testing in cattle, but does not have specific guidelines for small ruminants (sheep and goat). BTB is increasingly reported in small ruminants in European countries, and although usually low in prevalence it is nonetheless associated with severe pathology [8-11].

In many developing countries, small ruminants are the "poor man's cow", providing milk but also meat, hides and wool [12]. In rural traditional small holders in Ethiopia, small ruminants are important livestock components and are herded together with cattle during the day, whereas at night they are usually kept inside poorly ventilated farmer's houses (round hut made of mud and timber and thatched roof) for protection, thus having daily close contact to cattle and humans [13]. Such husbandry practices are epidemiologically important for potential disease transmission at the human-animal interface.

The prevalence of BTB in cattle in Meskan Woreda (*district*) was reported to be as high as 7.9% [13,14]. Tschopp et al. showed also that keeping small ruminants with cattle herds was associated with higher numbers of positive BTB reactors in cattle [13]. However, there are no published reports on confirmed *M. bovis *infection in small ruminants so far in Ethiopia.

Furthermore, tuberculous lymphadenitis (TBLN) in humans was shown to be a major problem in Meskan Woreda and was reported in 40% of all diagnosed tuberculosis patients in Butajira hospital [15]. TBLN can be caused by ingestion of raw animal products containing *M. bovis *[16]. The authors noticed, while conducting BTB surveillance in cattle in the Meskan Woreda, that farmers often mentioned members in their household having enlarged superficial lymph nodes characteristic for TBLN. However, these patients rarely went to the Butajira hospital and rather sought help in traditional medicine (Tschopp R., unpublished data).

The aim of this study was to gain better understanding of BTB at the cattle-small ruminant-human interface in the Meskan Woreda. The authors went back to the same villages where they had previously surveyed cattle for BTB [14], traced back the owners involved in the previous study to test their goats and sheep. In addition, TBLN prevalence was investigated in people in these villages.

## Methods

### Study site

**Figure 1:**
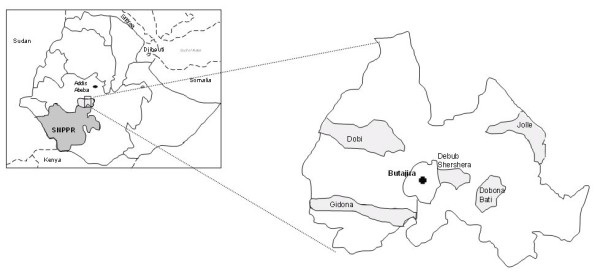
**Map illustrating the sample sites (light grey surfaces represent the Kebeles within Meskan Woreda, where samples were collected and Butajira represent the main town)**.

The study was conducted in Meskan Woreda, Gurage Zone, in the Southern Nations, Nationalities and Peoples Regional State Region (SNNPR) in Ethiopia. The area is located 130 km south of Addis Ababa, with Butajira being the main town (Figure 1). It has varying climates zones from arid dry lowland areas around 1500 m altitude to cool mountainous areas above 2000 m. The main wet season occurs between June and October while the remaining months are predominantly dry. All farmers included in the study were mixed crop-livestock smallholders from the Gurage and Silti ethnic groups.

### Study design

The authors conducted in 2007 and 2008 a stratified cluster sampling proportional to the size of the cattle population in Kebeles, where villages were considered as clusters based on calculated intra-class correlation coefficients [14]. A list of all Kebeles (smallest administrative unit) within the Woreda as well as a list of all villages within each Kebele was obtained from the Woreda agricultural office in Butajira. Five Kebeles were then randomly selected using random numbers generated in Microsoft Excel^®^; Villages were selected randomly and their number was proportionally to their cattle number within Kebeles.

The calculation of the sample size was done using formulas of Bennet et al. [17] as described in Tschopp et al. [13]. The authors considered herds belonging to individual owners but they were also regrouped into one single "village herd" for each village, since animals were kept communally during the day. Animals younger than 6 months of age, in late stage pregnancy and clinically sick were not included in the study. All tested animals were dewormed on the reading day for tuberculin results with Albendazol boli (Ashialben 2500 for cattle and Ashialben 600 for sheep and goats, Ashish Life Science PVT, Mumbai, India).

### Intradermal tuberculin testing in cattle

We applied the comparative intradermal tuberculin test (CIDT) in all cattle using both avian and bovine purified protein derivates (PPD) supplied by the Veterinary Laboratories Agency, Weybridge, UK. Intradermal injections of 0.1 ml (2,500 IU/ml) bovine PPD and 0.1 ml (2,500 IU/ml) avian PPD were made in two shaved sites, 12 cm apart from each other in the middle neck region, after having recorded skin thickness with a caliper. Skin thickness was measured again at both injection sites after 72 hours. The reaction at each site was derived as the difference of the skin thickness after 72 hours minus before injection. The data was analyzed using two different diagnostic thresholds to define positive test results: 1) an animal was considered positive if the bovine minus the *M. avium *reaction was greater than 2 mm [18,19] and 2) an animal was considered positive if the bovine minus the *M. avium *reaction was greater than 4 mm (OIE definition). The > 2 mm cut-off has been shown in previous studies to be more suitable for testing zebu cattle [18], but it has not yet been registered as an official cut-off by the OIE, hence the reason for presenting both cut-offs. A village-herd was considered positive if at least one positive reactor was present.

To assess the prevalence of *M. avium*, we defined arbitrarily *Mycobacterium avium *complex (MAC) positive reactors by assessing the skin reaction on the avium site alone as the difference between the initial avium PPD injection and the 72-hour reaction being greater than 4 mm.

### Assessment of BTB in small ruminants

Small ruminants were tested in the same village where the cattle survey took place. However, due to local political insecurities in Dobi Kebele at the time of the study, small ruminants were tested only in 4 Kebeles (Jolle, Dobona Bati, Gidona Aborat and Debub Shershera) from February to September 2009. Only farmers who had had their cattle tested in the last 2 years were included in the study for testing their goats and sheep.

Contrary to the cattle testing, no randomized procedure and no restriction in number of animals to be tested applied for the small ruminant testing. This was due to the limited number of eligible owners and the general low number of small ruminants in the villages.

Same testing, dosage and interpretation procedure applied as with cattle (described above) with the exception that PPD injections were performed on each side of the middle neck rather than on the same side like in cattle.

Upon agreement, CIDT positive animals were purchased from the owner, and slaughtered with subsequent post-mortem examination. Mediastinal, submandibular and mesenteric lymph nodes were collected under sterile conditions. Samples were stored in sterile saline buffer solution in universal tubes, kept and transported in ice-box to the Armauer Hansen Research Institute (AHRI) in Addis Abeba, where they were frozen at minus 20°C until further processing.

In addition, questionnaires with closed questions were filled in with each tested small ruminant owner. Questions were related to husbandry, livestock species present in the farm, history of diagnosed and/or treated TB/EPTB in the household, and consumption practice of goat and sheep milk.

### Human FNA sampling

The human study was carried out from April to December 2008 and received ethical clearances from the Institutional Ethical Review Committee (AHRI/ALERT; Ref. P004/04) and from the National Ethical Review Committees (NERC; Ref. RDHE/178-71/2006). In four Kebeles in which livestock were PPD tested, chairmen informed the population of their Kebele to meet in a defined communal area at a specific date if any of the individuals had swollen lymph nodes (locally called *Anget Biret*). Since no house to house survey was done, we do not know the underreporting in a Kebele. Only individuals originating from villages where PPD testing was done were included in this study. Informed consent was obtained from all participants. All individuals were then examined clinically by a trained physician. If TB-lymphadenitis was suspected, and after given informed written consent, a fine needle aspirate (FNA) sample was collected under sterile conditions from the patient's swollen lymph node using a 21-gauge needle. The content of the aspirate was split into two; one part was used for slide smears that were air dried in the field and subsequently stained at AHRI with Ziehl-Neelsen (ZN) for AFB agents and with Wright stain for cytological assessment. The slides were assessed by a trained pathologist. The other part of the aspirate was collected into Falcon tubes containing phosphate buffered saline (PBS) solution for culture.

Questionnaires were filled in with each patient. Questions included personal information (age, sex, occupation, education, marital status and religion), exposure to potential risk factors for BTB (contact with livestock, consumption of raw milk), clinical data of the patient (fever, cough, night sweat, poor appetite, weakness, weight loss, previous antibiotic or anti-TB treatment), data on physical examination of the swellings (location, number, size, mobility, texture, and presence or absence of draining sinuses or overlying scars) and finally whether or not livestock were PPD tested in their household.

All laboratory confirmed TB cases (cytology, culture) were reported back to the physician in Butajira involved in the study. Patients were then traced back in the villages and treatment initiated.

### Laboratory analysis

#### Culture

Homogenization and neutralization of lymph node tissue from skin test positive animals and FNAs from humans were done according to standard methods. Sediments were inoculated on Loewenstein-Jensen (LJ) media with and without pyruvate and on Middlebrook 7H11 media supplemented as previously described [20]. The tubes were incubated at 37°C for up to eight weeks and bacterial growth was examined weekly. Cultures were considered negative if no colony growth was detected after 8 weeks. Cultures that showed colony growth were stained with Ziehl-Neelsen to assess acid fast positive (AFB) isolates.

#### Molecular typing

DNA extraction from AFB positive colonies was performed according to Zwadyk et al. by the lysis method: one loop-full of bacteria from AFB positive cultures were suspended with reference strains of *M. tuberculosis *and *M. bovis *in 400 μl of 1xTE (Tris ethylene di-amine tetra acetic acid) and incubated for one hour at 80°C [21]. AFB positive cultures were stored at -80°C as 20% glycerol stocks. Heat-killed AFB positive samples were assessed by multiplex PCR for the presence or absence of RD4 and RD9 as described by Parsons et al. [22]. Isolates belonging to the *M. tuberculosis *complex were analysed by standard spoligotyping method as previously reported [23] using home-made membranes [24]. Spoligopatterns were analyzed using SPOLDB 4 database [25].

### Statistical analysis

Data was double entered in Microsoft Access and the two tables compared with EpiInfo (version 3.3.2). Data analysis was performed with the software package STATA 10.1 (StataCorp, Texas, USA).

Logistic regression models with random effect on village were used for the analysis of BTB prevalence in cattle and small ruminants as well as to assess risk factors associated with positive reactors. All results were expressed in odds ratio, 95% confidence interval of the odds ratio and p-values based on the likelihood-ratio test. Patient data was analyzed for associations with clinical features and with positivity in small ruminants and cattle.

## Results

### Tuberculin reactors in cattle

A total of 1214 cattle were tested in five Kebeles. Results of the skin testing for both the 4 and 2 mm cut-offs are shown in Table 1. Overall individual prevalence was 1.6% (CI: 0.9; 2.8%) and 6.8% (CI: 5.4; 8.5%) for the 4 mm and the 2 mm cut-off respectively. All villages had at least one positive reactor when using the 2 mm cut-off (100% herd prevalence), and 14 out of 21 villages were positive with the 4 mm cut off (66.7% herd prevalence). MAC prevalence varied between 0.9% in Jolle and 6.8% in Dobona Bati (Table 1).

**Table 1:** Prevalence in cattle and shoats in the different Kebeles, calculated with logistic regression with random effect on villages

			Debub Shershera	Dobona Bati	Gidona Aborat	Jolle	Dobi	Overall
Elevation (meter)			1960-1980	1800-1830	2090-2145	1800-1885	2140-2170	1800-2170
								
Number of cattle tested			167	177	295	297	278	1214
								
Number of sheep and goats tested			44	91	130	141	NA	406
	Number (%) of sheep		19 (43)	73 (80)	121 (93)	130 (92)	NA	343 (84.5)
	Number (%) of goats		25 (57)	18 (20)	9 (7)	11 (8)	NA	63 (15.5)
								
Prevalence (LCL-UCL^†^) in %	Cattle	4 mm cut off	1.19 (0.29; 4.65)	2.25 (0.85; 5.86)	1.35 (0.50; 3.55)	2.11 (0.48; 8.82)	1.07 (0.34; 3.29)	1.59 (0.88; 2.85)
		2 mm cut off	5.98 (3.25; 10.76)	9.60 (6.05; 14.90)	5.98.0 (2.970; 11.65)	6.73 (4.38; 10.20)	5.75 (3.55; 9.18)	6.76 (5.36; 8.49)
								
	Sheep and goats	4 mm cut off	0	0	0	0	NA	0
		2 mm cut off	0	0	1.16 (0.08; 13.55)*	0.70 (0.09; 4.85)**	NA	0.41 (0.03-5.15)
								
Prevalence MAC (LCL-UCL) in %	Cattle		6.58 (3.68; 11.50)	6.78 (3.89; 11.56)	4.74 (2.82; 7.85)	0.96 (0.10; 8.61)	3.95 (2.20; 7.00)	4.44 (3.42; 5.76)
	Sheep and goats		2.27 (0.31; 14.44)	0	0	0	NA	0.24 (0.03; 1.72)

^†: ^LCL-UCL stands for lower confidence limit and upper confidence limit

*: 2 positive animals (1 sheep, 1 goat)

**: 1 positive animal (1 sheep)

### Tuberculin reactors in small ruminants

A total of 406 small ruminants belonging to 134 owners in 12 villages from 4 Kebeles were tested for BTB using the CIDT. Goats (N = 63; 15.5%) were outnumbered by the sheep population tested (N = 343; 84.5%). Overall, females accounted for 77% of the animals and 97.3% of all tested animals were in good body condition.

None of the tested small ruminants were BTB reactors with the > 4 mm cut-off. With the > 2 mm cut-off, 3 animals (2 sheep and 1 goat) were CIDT positive, thus resulting in an individual prevalence of 0.41% (CI: 0.3% - 5.1%) and a herd prevalence of 25%.

One goat and one sheep were from the same village, but from different owners, whereas the second positive sheep was from another village.

Only one goat was reactor for MAC (0.25%). Results of the prevalence in the small ruminants are presented in Table 1.

In the univariate analysis, goats were more at risk than sheep for being positive reactor (OR: 5.2; 95%OR: 0.23; 114.2) as were male than female animals (OR: 1.6; 95%OR: 0.13; 18.0). These results were however not statistically significant.

### Descriptive epidemiology of the risk factors

Out of the 134 owners with tested small ruminants (96% kept sheep and 27% goats), 127 had had their cattle previously tested. Six of these owners (4.7%) and 28 (22%) had positive cattle reactor with the > 4 mm and > 2 mm cut-off, respectively. Cattle of the owners with PPD positive sheep and goats were all BTB negative. But, the cattle herd prevalence in the two villages with small ruminant reactors was 5.2% and 8.5% respectively. No further analysis of risk factors (e.g. purchasing, housing, herding) have been done since only 3 out of 406 animals were positive for BTB.

Mean number of sheep by owner was 4. Half of the goat owners possessed only 1 animal.

Eighteen farmers (13%) did not buy any small ruminants in the last five years and used their own animals for reproduction. Seventy-seven (57%) owners, including the three owners with positive shoats purchased animals over two years ago at the local market.

Hundred-and-eleven respondents (83%) kept their small ruminants inside their house during the night, whereas 23 (17%) kept them in a closed shed outside the living house. Seven (5%) farmers stated that they regularly drink milk from small ruminants, mainly goat milk. Five (3.7%) boiled the milk together with their coffee whereas 2 (1.5%) usually drank it raw.

Fifteen farmers (11%) had a family member treated for pulmonary tuberculosis at the Butajira hospital and 12 (9%) had been clinically diagnosed with EPTB in the last five years.

### Molecular analysis of isolates from small ruminants

One goat, being reactor with the > 2 mm cut-off was purchased, slaughtered and lymph node samples collected for culture and molecular typing. *M. tuberculosis *(SIT837) was isolated from the goat specimens (Table 2).

**Table 2:** Spoligotyping patterns of *M.tuberculosis *complex strains isolated from humans and a goat in Meskan Woreda (Ethiopia)

Spoligotype**	Host	Isolates number	1	2	3	4	5	6	7	8	9	10	11	12	13	14	15	16	17	18	19	20	21	22	23	24	25	26	27	28	29	30	31	32	33	34	35	36	37	38	39	40	41	42
43SIT53	Human	3	■	■	■	■	■	■	■	■	■	■	■	■	■	■	■	■	■	■	■	■	■	■	■	■	■	■	■	■	■	■	■					■	■	■	■	■	■	■
SIT26	Human	1	■	■	■					■	■	■	■	■	■	■	■	■	■	■	■	■	■	■												■	■	■	■	■	■	■	■	■
SIT36	Human	2	■	■	■	■	■	■	■	■	■	■	■	■		■	■	■	■	■	■	■	■	■	■	■	■	■	■	■	■		■					■	■	■	■	■	■	■
SIT149	Human	1	■	■	■	■	■	■	■	■	■											■	■	■	■	■	■	■	■	■	■	■	■					■	■	■	■	■	■	■
unique*	Human	2	■	■	■	■	■	■	■	■	■	■	■	■		■	■	■	■	■		■	■	■	■	■	■	■	■	■	■		■					■	■	■		■	■	■
SIT837	goat	1	■	■	■	■	■	■	■	■	■	■	■	■	■	■	■	■	■	■	■	■	■	■	■	■	■	■	■				■					■	■	■		■	■	■

** SIT no: Spoligo-International-Typing

* this unique strain could not be identified in the SIT database

### Human FNA

#### Clinical description

A total of 127 individuals were screened by a physician in 4 Kebeles. FNAs were taken from a total of 33 patients with clinical diagnosis of lymphadenitis. The majority were females (67%). Sixty-one percent were between 19 and 45 years old. Children under 10 years accounted for 9% of the total FNAs, whereas each 15% of the patients were between 10 and 18 years and over 45 years old.

Clinical signs accompanying lymphadenitis were: weakness (N = 27; 82%), fever (N = 21; 64%), body weight loss (N = 21; 64%), loss of appetite (N = 18; 54.5%), night sweat (N = 15; 45.5%) and cough (N = 8; 24%).

The majority of the aspirates were from cervical lymph nodes (N = 15; 45.5%) followed by sub-mandibular lymph nodes (N = 8; 24.2%). Further aspirates were taken from axillar (N = 3; 9%), suprascapular (N = 3; 9%), inguinal (N = 2; 6%), post-auricular (N = 1; 3%) lymph nodes and one breast mass. A single swollen lymph node was seen in the majority of the cases (N = 22; 67%) when compared to multiple nodes.

There was no relationship between age or sex and location of swollen lymph node (Fisher's exact test: p = 0.19 and 0.76, respectively) and between age or sex and number of nodes (Fisher's exact test: p = 0.42 and 0.22, respectively).

Sixty-four percent of the patients stated that lymph node swelling were older than 12 months.

#### Laboratory results

Based on the cytology results, 12 were confirmed TBLN cases (caseous necrosis with degenerative cells), 14 (44%) were non-TBLN (non-specific lymphoid hyperplasia) and 6 slides (18.5%) could not be used as diagnostic due to high levels of hemorrhages.

Out of the 33 cultures, 9 showed AFB positive colony growth (27.3%). All were shown to be *M. tuberculosis *by deletion typing (RD 9). Further spoligotyping showed 5 distinct patterns (Table 2).

The comparison between cytology and culture results showed that 1 non-TBLN and 3 non-diagnostic cytology results were TB positive on culture and PCR. Furthermore, 7 out of the 12 (58%) TB positive cytology results were TB negative on culture.

#### Characteristics of patients with lymphadenitis

Twenty-four lymphadenitis patients (73%) had livestock. Six of them had their cattle and 9 had their small ruminants tested for BTB. All these tested animals were PPD negative.

Raw milk was regularly consumed by 17 (51.5%) patients. Three lymphadenitis patients (9%) stated to have regular contact with diagnosed TB patients; however none of the 3 had confirmed TBLN. No TBLN was seen in patients who were younger than 12 or older than 40 years. Results of the univariate logistic regression analysis are shown in Table 3.

**Table 3:** Univariate analysis for the 9 PCR confirmed TBLN patients among all lymphadenitis patients (logistic regression analysis using likelihood ratio test)

Risk factors	Categories	Number of lymphadenitis patients	Number PCR confirmed TBLN	OR	p-value	95% CI OR
Sex	Female	22 (67%)	4	1		
	Male	11 (33%)	5	3.75	0.11	0.75; 18.7
						
Age class	< 15 years	6 (18%)	2	1		
	15-35 years	20 (61%)	6	0.8		0.12; 6.01
	> 35 years	7 (21%)	1	0.33	0.65	0.02; 5.02
						
Raw milk consumption	Yes	17 (51.5%)	5	1		
	No	16 (48.5%)	4	0.8	0.77	0.17; 3.72
						
						
Livestock in household	Yes	24 (73%)	7	1		
	No	9 (27%)	2	0.7	0.68	0.11; 4.2)
						
						
Education level	No education	25 (76%)	5	1		
	Primary school	8 (24%)	4	0.109	4	0.73; 21.8
						
Contact with TB patient	Yes	3 (9%)	0			
	No	30 (91%)	9	NA	NA	NA

## Discussion

Bovine tuberculosis was endemic in cattle in the study area with 100% herd prevalence (> 2 mm cut-off). Individual prevalence using the same cut-off varied between Kebele between 5.7 and 9.6%. However, despite continuous close contact with potential BTB cattle, the individual tuberculine-positivity prevalence in small ruminants was very low (overall 0.4%). This is in line with findings for instance in Ireland [9], Pakistan [12], and New-Zealand [26]. It was long suggested that small ruminants, especially sheep are more resistant to bovine tuberculosis. In our study, the odds ratio for being positive reactor was 5.2 for goats. Although BTB prevalence in small ruminants remain generally low, studies in Spain have shown high prevalence of BTB in goats [27]; Experimental studies have also demonstrated that goats are susceptible to develop BTB (positive CIDT reaction and lesions) when inoculated with *M. bovis *[28]. *M. bovis *was recently repeatedly, though sporadically, isolated not only from goats but also from sheep showing severe pathology of the disease [10,11,29]. Various suggestions were made to explain the low prevalence usually seen in sheep and goats. Sharp stated a lack of opportunity for infection in sheep since flocks are usually kept extensively and tend to stay together and not mix with cattle [30]. Although this might apply for industrialised countries, in our study small ruminants had daily close and intensive contact with cattle in communal herding practice on often overstocked pasture due to the scarcity of grazing land. This husbandry practice- as seen in most parts of Africa- gives plenty of opportunity for disease transmission and still the prevalence in small ruminant was very low. In goats, the feeding behavior is possibly playing a role since they are predominantly browsers and not grazers and have thus less access to contaminated pastures. Turn-over of animals also influences BTB prevalence as seen in cattle [13] and small ruminants [12]: the older the animal and the longer it stays in a herd, the more likely it is exposed to the disease. Life span of small ruminants in Ethiopia is short, since they are primarily kept for meat.

It is known that the sensitivity of the CIDT in small ruminants varies between settings, for example from 44.6% [27] to 83.7% [31]. Up to date, there are no official standards for skin testing in small ruminants and the testing and result interpretation follows very much the cattle OIE standards with some individual author adaptations when it comes to cut-off used. Anecdotally, the authors noticed often severe skin reaction after PPD injections, especially in goats (erythema, pain, small necrotic foci at the injection site). In addition, Alvarez et al. showed that paratuberculosis (*M. avium subsp paratuberculosis*) interferes with the CIDT result and that the CIDT alone becomes unreliable when paratuberculosis is prevalent in small ruminants [32]. To this date, no data exist on paratuberculosis prevalence in cattle or small ruminants in Ethiopia and thus the authors cannot exclude the possibility of such interference. However, MAC prevalence was very low suggesting minimal involvement of paratuberculosis in our study.

Finally, Javed et al. showed a breed related BTB prevalence in sheep in Pakistan [12]. Thus some breed might be naturally more resistant to the disease or less reacting to PPD More research is warranted in breed genetics in Ethiopia and their susceptibility to BTB.

Some authors suggest that small ruminants act only as amplifier hosts and cannot maintain the disease in a herd [33]. BTB in small ruminant would become a problem only when they are in close contact with cattle with high disease prevalence [9]. However, reports show that they are susceptible to BTB, that the respiratory tract is often affected and that therefore they remain a potential source of infection for other animals or humans. In our study, 83% of the farmers kept their sheep and goat inside their house at night and 5% drank regularly goat milk. This highlights the potential of zoonotic transmission.

Of all farmer families whose livestock were tested, 9% were diagnosed with lymphadenitis in the last five years, suggesting a possible animal source of infection through consumption of infected raw animal product. However, no *M. bovis *was isolated from the TBLN patients. Furthermore, all TBLN positive people had PPD negative animals. All FNA samples yielded *M. tuberculosis*, the typical human pathogen. Molecular analysis of sampled lymph nodes from a PPD positive goat in our study showed an infection with *M. tuberculosis *as well and not with *M. bovis*. Unfortunately, we did not have any isolates from positive cattle in these villages. However, Berg et al. isolated *M. tuberculosis *in 2 out of 18 cattle (11%) during an abattoir survey in Butajira [24], which also slaughters cattle originating from our study site. *M. tuberculosis *circulates in the cattle and goat population in the area. The question is whether humans are a source of infection for livestock through close contact with infected animals (sharing houses at night) or are there livestock (cattle-goat-sheep) adapted *M. tuberculosis *strains. Interestingly, the *M. tuberculosis *spoligotype isolated from the goat differed from the spoligotypes isolated from humans in this study. In recent years, an increasing number of *M. tuberculosis *was isolated from cattle [24,34-36]. The pathogen was also isolated from milk [35] suggesting a risk of infection of humans through consumption of raw milk.

Five different spoligopatterns were isolated from humans, some of which are common all over Ethiopia and others that seem to be more prevalent in Butajira (SIT 36, SIT 26) (AHRI database, unpublished data).

Diagnosing properly patients with TB is crucial. Our study highlighted that TBLN diagnosed only on the basis of smear microscopy is unreliable, since it missed 33.3% of patients truly having TB. The high number of missed TB cases among TB negative smear microscopy is a concern raised also by other authors [15,37]. FNA cytology in TBLN is a relatively easy, useful and a major diagnostic method especially in resource poor countries, with a sensitivity and specificity reaching 76% and 88% respectively [38]. This method has pitfalls mainly related to poor technique in making readable smears and in aspiration. The latter can also lead to poor Mycobacteria yield in culture originating from FNAs and thus contribute to false negative culture results. Since the sensitivity of smear diagnosis alone is poor, it is essential that diagnosis of TB is further supported with culture and molecular methods.

## Conclusion

In conclusion, we showed that tuberculosis was rare in small ruminants despite them sharing pastures with BTB positive cattle. The public health and economic concerns that result from *M. tuberculosis *circulating between livestock and humans at this interface should warrant more research in identifying the role played by *M. tuberculosis *in animals, its transmission to humans and why small ruminants seem to be rather resistant to *M. bovis *in Ethiopia.

## Competing interests

The authors declare that they have no competing interests.

## Authors' contributions

RT designed the study, and carried out sample collections with inputs from KB, AA, DJ, and JZ. MH, KB, RI, EH, JH and RF were responsible for various laboratory analysis. RT and ES conducted the data analysis. RT drafted the final manuscript. All authors read and approved the final manuscript.

## Pre-publication history

The pre-publication history for this paper can be accessed here:

http://www.biomedcentral.com/1471-2334/11/318/prepub
